# Structure-based engineering of Tor complexes reveals that two types of yeast TORC1 produce distinct phenotypes

**DOI:** 10.1242/jcs.261625

**Published:** 2024-02-28

**Authors:** Yoshiaki Kamada, Chiharu Umeda, Yukio Mukai, Hokuto Ohtsuka, Yoko Otsubo, Akira Yamashita, Takahiro Kosugi

**Affiliations:** ^1^ Interdisciplinary Research Unit, National Institute for Basic Biology (NIBB), National Institutes of Natural Sciences (NINS), Okazaki, Aichi, 444-8585, Japan; ^2^ Basic Biology Program, SOKENDAI (The Graduate University for Advanced Studies), Hayama, Kanagawa, 240-0193, Japan; ^3^ Department of Frontier Bioscience, Nagahama Institute of Bio-Science and Technology, Nagahama, Shiga, 526-0829, Japan; ^4^ Laboratory of Molecular Microbiology, Graduate School of Pharmaceutical Sciences, Nagoya University, Nagoya, Aichi, 464-8601, Japan; ^5^ Research Center of Integrative Molecular Systems (CIMoS), Institute for Molecular Science (IMS), National Institutes of Natural Sciences (NINS), Okazaki, Aichi, 444-8585, Japan; ^6^ Exploratory Research Center on Life and Living Systems (ExCELLS), National Institutes of Natural Sciences (NINS), Okazaki, Aichi, 444-8585, Japan; ^7^ Molecular Science Program, SOKENDAI (The Graduate University for Advanced Studies), Hayama, Kanagawa, 240-0193, Japan; ^8^ Precursory Research for Embryonic Science and Technology (PRESTO), Japan Science and Technology Agency, Kawaguchi, Saitama, 332-0012, Japan

**Keywords:** Lifespan, Protein complex, Protein engineering, Target of rapamycin, Yeast

## Abstract

Certain proteins assemble into diverse complex states, each having a distinct and unique function in the cell. Target of rapamycin (Tor) complex 1 (TORC1) plays a central role in signalling pathways that allow cells to respond to the environment, including nutritional status signalling. TORC1 is widely recognised for its association with various diseases. The budding yeast *Saccharomyces cerevisiae* has two types of TORC1, Tor1-containing TORC1 and Tor2-containing TORC1, which comprise different constituent proteins but are considered to have the same function. Here, we computationally modelled the relevant complex structures and then, based on the structures, rationally engineered a Tor2 mutant that could form Tor complex 2 (TORC2) but not TORC1, resulting in a redesign of the complex states. Functional analysis of the Tor2 mutant revealed that the two types of TORC1 induce different phenotypes, with changes observed in rapamycin, caffeine and pH dependencies of cell growth, as well as in replicative and chronological lifespan. These findings uncovered by a general approach with huge potential – model structure-based engineering – are expected to provide further insights into various fields such as molecular evolution and lifespan.

## Teaser

**Highlighted Article:** Structure-based engineering uncovers functional differences for a protein complex: two types of yeast TORC1 have distinct functions.

## INTRODUCTION

**Fig. 1.**
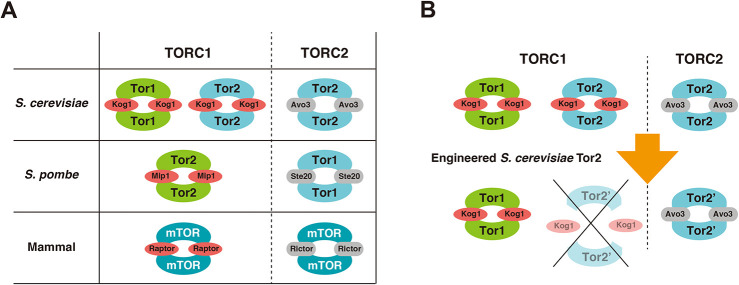
**Strategy to compare two types of TORC1 in *S. cerevisiae*.** (A) Orthologue proteins that constitute TORC1 and TORC2 in several species. Only *S. cerevisiae* has two types of TORC1, namely Tor1-TORC1 and Tor2-TORC1. (B) Tor2-TORC1 in *S. cerevisiae* is deleted by engineering Tor2 to maintain the ability to bind with Avo3 and to lose the ability to bind with Kog1. In this figure, Lst8 is omitted. The engineered Tor2 is indicated as Tor2′.

Many proteins assemble into complex states within cells, and a substantial proportion of these complexes exhibit various combinations of constituent proteins to proficiently exert their functions in the appropriate spatiotemporal context. Target of rapamycin (Tor), an evolutionarily conserved protein kinase, plays a pivotal role in eukaryotic cell signalling pathways. Tor kinases respond to changes in the extracellular environment, such as changes in nutritional status, and are associated with various diseases and the regulation of lifespan (Liu and Sabatini, 2020; Loewith and Hall, 2011). Tor kinases form two distinct complexes, Tor complex 1 and Tor complex 2 (TORC1 and TORC2, respectively; Loewith et al., 2002). TORC1 and TORC2 comprise several different constituent proteins, which results in the two complexes exerting different functions (Fig. 1A) (Liu and Sabatini, 2020; Loewith and Hall, 2011). In *Saccharomyces cerevisiae*, the Tor1 serine/threonine protein kinase assembles into TORC1 with the main partner Kog1 [the yeast counterpart of Raptor (RPTOR)] and other partner proteins such as Lst8. Another *S. cerevisiae* kinase protein, Tor2, not only assembles into TORC1 but also forms TORC2 with the main partner Avo3 (also known as TSC11; the yeast counterpart of Rictor) and other proteins, including Lst8, Avo1 and Avo2 (Loewith et al., 2002). Thus, in *S. cerevisiae* there are two types of TORC1, containing either Tor1 or Tor2 (hereafter referred to as Tor1-TORC1 and Tor2-TORC1, respectively), whereas other species, such as mammals and the fission yeast *Schizosaccharomyces pombe*, have only one type of TORC1 (Fig. 1A) (Otsubo et al., 2017).

To the best of our knowledge, functional differences between Tor1-TORC1 and Tor2-TORC1 have not yet been studied. Tor2-TORC1 has been ignored because the amount of Tor2-TORC1 in cells is lower than that of Tor1-TORC1 (Loewith et al., 2002; Reinke et al., 2004). Moreover, the functions are thought to be the same because Tor1 is a homologue of Tor2 with high sequence identity (66.2%) and the same ligands are phosphorylated by both Tor1-TORC1 and Tor2-TORC1 (Kunz et al., 1993). However, these complexes exhibit several interesting assembly properties. Tor2 assembles into both complex states, whereas Tor1 does not assemble into TORC2. Furthermore, there is no chimeric TORC1 that contains both Tor1 and Tor2; in a TORC1 complex, only either Tor1 or Tor2 is included (Loewith et al., 2002; Reinke et al., 2004; Takahara et al., 2006).

TORC1 and TORC2 activity in *S. cerevisiae* is essential for cell growth (Loewith and Hall, 2011). *TOR1* deletion is not lethal because only Tor1-TORC1 is deleted and Tor2-TORC1 remains. The *tor1*Δ strain has been the subject of intense investigation. It has been shown that the cell lifespan of *tor1*Δ cells is extended (Kaeberlein et al., 2005) because the partial inhibition of TORC1 mimics calorie restriction, an important factor of longevity (Lin et al., 2000). Inhibition of TORC1 using drugs, such as rapamycin or Torin-1, also leads to longevity in various organisms, including yeast, nematodes, flies and rodents. (Dabrowska et al., 2022; Folch et al., 2018; Harrison et al., 2009; Martinez-Miguel et al., 2021; Ohtsuka et al., 2021b; Rodríguez-López et al., 2020). For lifespan, Tor1-TORC1 is expected to have a function similar to that of TORC1 in other species. However, the function of Tor2-TORC1 is unclear. *TOR2* deletion is lethal because only Tor2 assembles into TORC2 (Kunz et al., 1993; Loewith et al., 2002); this could be one of the reasons why Tor2-TORC1 has never been studied.

Studies on Tor complexes by domain exchange have not revealed the differences between Tor1-TORC1 and Tor2-TORC1, and have instead focused on identifying important interactions for assembling complex states (Hill et al., 2018; Tsverov et al., 2022). High-resolution three-dimensional structures determined using X-ray crystallography or cryo-electron microscopy (cryo-EM) can uncover important interactions within complexes. Moreover, a comparison of the three-dimensional structures of *S. cerevisiae* Tor1-TORC1 and Tor2-TORC1 might provide clues about their functions. However, high-resolution structures for these complexes have not yet been obtained.

Recently, remarkable developments in computational protein structure prediction and protein design methods have been achieved (Baek et al., 2021; Dauparas et al., 2022; Huang et al., 2016; Jumper et al., 2021). Using computational methods, native proteins have been redesigned and their functions have been successfully controlled. For example, artificial activation or inactivation of G-protein-coupled receptors and cyclic GMP–AMP synthase by state-targeting stabilisation have been reported (Chen et al., 2020; Dowling et al., 2023). We have also previously controlled the concerted function – rotation – of a rotary molecular motor, the vacuolar-type ATPase (V-ATPase) V_1_ domain, using a novel approach based on computational protein design methods (Kosugi et al., 2023). Here, based on predicted structural models for protein complexes whose experimental structures are unavailable, we engineered a constituent protein to change the pattern of possible combinations and attempted to uncover the biological functions of a protein complex in cells.

In this study, using structure-based engineering, we designed a Tor2 mutant protein that could not form TORC1 but could form TORC2 (Fig. 1B). Mutant strains of *TOR2* showed differences in several phenotypes when compared with the *tor1*Δ strain, including rapamycin, caffeine and pH dependencies of cell growth, as well as replicative and chronological lifespans. These results reveal that several characteristics of Tor2-TORC1 are different from those of Tor1-TORC1. Based on the differences in the roles of these two complexes, we propose new perspectives for research on molecular evolution and lifespan.

## RESULTS

### Structure-based engineering of Tor2 to abrogate Tor2-TORC1 assembly but retain TORC2 formation

**Fig. 2.**
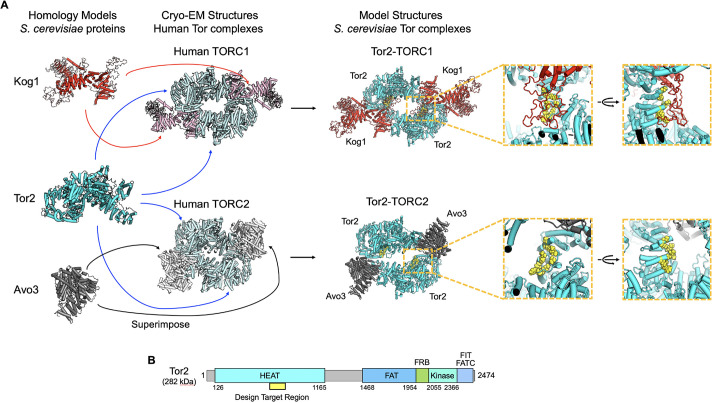
**Strategy to create and compare two model structures to find a mutation target region in Tor2.** (A) Model building protocol and model structures of Tor2-TORC1 (top) and TORC2 (bottom) (Lst8 is omitted). Homology models of each constituent protein were superimposed on the constituent proteins in cryo-EM structures of human Tor complexes. The amino acid residues in the design target region, with which Kog1 interacts but Avo3 does not, are shown as yellow spheres. (B) Design target region (yellow) indicated on the primary sequence of Tor2. The region is in the Tor2 HEAT domain. The positions of the FAT, FRB, kinase, FIT and FATC domains are also indicated.

**Fig. 3.**
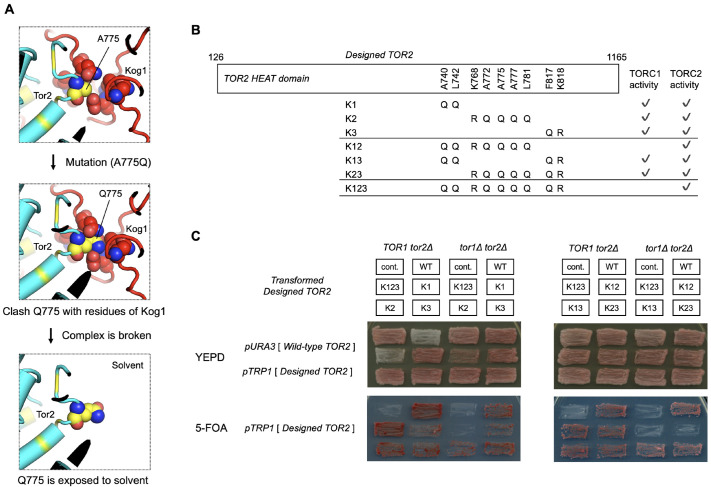
**Strategy to design Tor2 mutants to form TORC2 but not TORC1, and a cell-based assay for the redesigned Tor complexes with TORC2 function but no TORC1 function.** (A) Illustrative case of the A775 residue of Tor2 in the redesign strategy. A775 in the design target region was mutated to glutamine (Q775), which clashes with Kog1 and stabilises the resulting surface exposed to solvent. The design target residues in Tor2 (cyan) are shown in yellow. A775 (Q775) in Tor2 and the surrounding residues in Kog1 (red) are shown as spheres. (B) Mutation sites and amino acid substitutions of the designed Tor2 mutants. A summary of the cell-based assay in C is also shown. (C) Cell-based activity assay of Tor complexes using plasmid-based expression of the designed *TOR2* mutants as shown in B (K1, K2, K3, K12, K13, K23 and K123). Schematic (top) shows the strain background genotypes and the pattern of strain plating. Cont., control with expression of wild-type *TOR2* from a *URA3* marker plasmid only. WT, control with expression of wild-type *TOR2* from both *URA3* and *TRP1* marker plasmids. Cells were patched onto YEPD (middle) and 5-FOA (bottom) plates and incubated for 2 days at 30°C. On YEPD plates, wild-type *TOR2* is expressed from a *URA3* marker plasmid. On 5-FOA plates, *ura3^−^* cells are selected, and only expression of the designed *TOR2* from the *TRP1* marker plasmid remains. Tor2(K12) and Tor2(K123) have no TORC1 activity and retain TORC2 activity, as designed. Images are representative of two repeats.

To engineer Tor2 to not assemble into Tor2-TORC1 but to maintain TORC2 assembly, we focused on the interactions between Tor2 and the unique components of each complex – Kog1 for Tor2-TORC1 and Avo3 for TORC2. We aimed to eliminate the interaction of Tor2 with Kog1 and retain interaction with Avo3 by structure-based engineering. Therefore, reasonable structures for Tor2-TORC1 and TORC2 were required. However, high-resolution structures of Tor2-TORC1 and TORC2 from *S. cerevisiae* have not been reported, except for a recently resolved cryo-EM structure of the TORC1 inactive condensate, TOROID (Prouteau et al., 2023). Cryo-EM structures of human TORC1 and TORC2 (mTORC1 and mTORC2, respectively) have been reported at approximately 3.2Å resolution (Scaiola et al., 2020; Yang et al., 2017). Therefore, by superimposing homology models of each constituent protein (Tor2, Kog1 and Avo3) from *S. cerevisiae* on the human Tor complex structures, we computationally modelled a dimer of the Tor2 and Kog1 protein complex as Tor2-TORC1, and a dimer of the Tor2 and Avo3 protein complex as TORC2 (Fig. 2A). By comparing these two Tor2-TORC1 and TORC2 model structures, we found a design target region in Tor2 that interacts with Kog1 but not with Avo3 within the HEAT domain; note that other constituent proteins, namely Lst8 and Avo2, also do not interact with this region. This target region contacts loop structures of Kog1 that are not present in orthologues from other species, namely *S. pombe* and humans (the sequence alignments are shown in Fig. S1). Thus, these loop structures were expected to contribute to TORC1 assembly of *S. cerevisiae* Tor2. In the design target region of Tor2, nine amino acid residues (A740, L742, K768, A772, A775, A777, L781, F817 and K818) were selected. The design target residues in the model structures and the design target region in the primary sequence are shown in yellow in Fig. 2A and B, respectively. The target residues were mutated to clash with the characteristic loop region of Kog1 and to stabilise the surface exposed to solvent; hydrophobic residues were mutated to a larger and hydrophilic residue, glutamine, and charged residues were mutated to larger amino acids with the same charge (Fig. 3A). As shown in Fig. 3B, seven combinations of the mutations – named K1, K2, K3, K12, K13, K23 and K123 – were experimentally validated.

### The engineered Tor2 mutant maintains TORC2 activity but does not have TORC1 activity

Tor2-TORC1 and TORC2 activities of the seven Tor2 mutant strains were verified using a cell-based assay. The *TOR2* mutant sequences were cloned into a pRS314 vector containing the *TRP1* selection marker, and the resulting plasmids were transformed into *TOR1 tor2*Δ and *tor1*Δ *tor2*Δ strains harbouring a wild-type *TOR2* expression plasmid containing the *URA3* selection marker. The transformants were streaked onto complete medium plates (YEPD, control) or onto plates containing 5-fluoroortic acid (5-FOA) to select *ura3^−^* cells that had lost the wild-type *TOR2*-expressing *URA3* marker plasmid and harboured only the mutated *TOR2* plasmid. Growth on 5-FOA plates was used to evaluate the function of TORC2 alone (*TOR1 tor2*Δ strain) and of TORC1 and TORC2 (*tor1*Δ *tor2*Δ strain) (Fig. 3C) because the loss of either TORC1 or TORC2 activity is lethal for cells. The K1, K2, K3, K13 and K23 Tor2 mutant transformants grew as well as the wild-type Tor2 control (strain with expression of wild-type *TOR2* from both *URA3* and *TRP1* marker plasmids) on 5-FOA plates in both *TOR1 tor2*Δ and *tor1*Δ *tor2*Δ backgrounds (Fig. 3B,C). In contrast, the K12 and K123 transformants grew on 5-FOA plates only in the *TOR1 tor2*Δ background, indicating that these two *TOR2* mutants do not function as TORC1. These results suggest that the two strains, K12 and K123, exhibit activities as expected from the design.

**Fig. 4.**
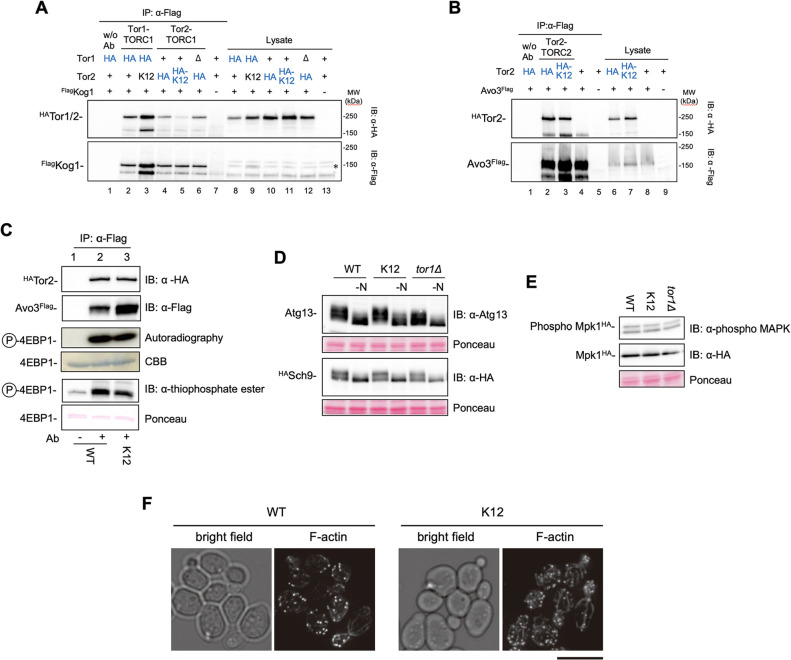
**The Tor2(K12) mutant almost loses TORC1-assembling ability and retains TORC2-assembling ability.** (A) Formation of Tor1-TORC1 and Tor2-TORC1. Flag-tagged Kog1 (^Flag^Kog1) was immunoprecipitated from cell lysates of wild-type, *tor2*(K12) and *tor1*Δ strains expressing either HA-tagged Tor1 (^HA^Tor1; lanes 8 and 9) or HA-tagged Tor2 (^HA^Tor2; lanes 10–12) using an anti-Flag (α-Flag) antibody [+, wild-type protein; Δ, *tor1*Δ; HA, HA-tagged protein; HA-K12, HA-tagged Tor2(K12); K12, untagged Tor2(K12)]. Co-precipitation of ^Flag^Kog1 with ^HA^Tor1 (lanes 1–3) and ^HA^Tor2 (lanes 4–6) in the immunocomplexes was detected by immunoblotting (IB) using anti-Flag and anti-HA (α-HA) antibodies. Lane 1 is an immunoprecipitation (IP) control without antibody (w/o Ab), and lanes 7 and 13 are IP controls without the tagged proteins. Tor2(K12) has reduced TORC1-assembling ability compared to that of Tor2 (compare lanes 4 and 5; 34%±10%, mean±s.d., *n*=4). Note that in the presence of Tor2(K12), a higher amount of Tor1-TORC1 was detected (lanes 3 and 9). ^Flag^Kog1 was marginally detected in cell lysates, partly because a non-specific band (*) overlapped the ^Flag^Kog1 band (lanes 8–13). The strains used were as follows: lanes 1, 2 and 8, YYK799; lanes 3 and 9, YYK1580; lanes 4 and 10, YYK1467; lanes 5 and 11, YYK1530; lanes 6 and 12, YYK1470; lanes 7 and 13, YYK1414. (B) Formation of Tor2-TORC2. Flag-tagged Avo3 (Avo3^Flag^) was immunoprecipitated from cell lysates of strains expressing the indicated forms of Tor2 (lanes 6–9), and co-precipitated ^HA^Tor2 contained in the immunocomplexes was detected (lanes 1–5). Lane 1 is an IP control without antibody, and lanes 5 and 9 are IP controls without the tagged proteins. Tor2(K12) retains the TORC2-forming ability (70%±20% of that of Tor2; mean±s.d., *n*=3). The strains used were as follows: lanes 1, 2 and 6, YYK1464; lanes 3 and 7, YYK1528; lanes 4 and 8, YYK1449; lanes 5 and 9, YYK1414. (C) *In vitro* protein kinase assay of TORC2. TORC2 was immunoprecipitated from strains expressing Avo3^Flag^ and either HA-tagged wild-type (WT) Tor2 or HA-tagged Tor2(K12) using an anti-Flag antibody (IP: α-Flag). Avo3 immunoprecipitation was confirmed by immunoblotting to detect Avo3^Flag^ (IB: α-Flag), and immunoprecipitation of the same amounts of TORC2 was estimated from the amount of ^HA^Tor2 protein (IB: α-HA). TORC2 kinase assays were performed using [γ-^32^P]ATP [autoradiography and Coomassie Brilliant Blue staining (CBB)] or ATPγS (IB: α-thiophosphate ester and Ponceau staining) as described in the Materials and Methods. TORC2 kinase activity was estimated as the phosphorylation activity towards 4EBP1 (P-4EBP1). The TORC2 kinase activity of Tor2(K12) is comparable to that of the wild-type Tor2. Data shown are representative of three independent experiments. The strains used were as follows: lanes 1 and 2, YYK1464; lane 3, YYK1528. (D) *In vivo* TORC1 kinase activities. Cells [wild-type, *tor2*(K12) and *tor1*Δ strains] harbouring plasmids expressing Atg13 (top) or HA-tagged Sch9 (^HA^Sch9; bottom) grown in YEPD at 30°C were treated by nitrogen starvation (−N) for 30 min, and the phosphorylation state of Atg13 and Sch9 was examined by immunoblotting (IB: α-Atg13 and IB: α-HA, respectively). Phosphorylation state is visible as a mobility shift. Ponceau staining is shown as a loading control. TORC1 in cells expressing Tor2(K12) has similar activity to that in cells expressing wild-type Tor2. Data shown are representative of three independent experiments. (E) *In vivo* TORC2 kinase activities. Cells [wild-type, *tor2*(K12) and *tor1*Δ strains] harbouring a plasmid expressing HA-tagged Mpk1 (Mpk1^HA^) grown in YEPD at 30°C were examined by immunoblotting for the phosphorylation state of Mpk1 (IB: α-phospho MAPK) relative to total Mpk1 (IB: α-HA). Ponceau staining is shown as a loading control. TORC2 activity of Tor2(K12)-TORC2 in cells is comparable to that of the wild type. Data shown are representative of two independent experiments. (F) Localisation of F-actin in the *tor2*(K12) mutant. WT and *tor2*(K12) mutant cells were grown in YEPD at 30°C and processed for F-actin staining. Bright-field images are shown on the left. Scale bar: 5 µm. Images are representative of three or four repeats.

To further characterise the K12 and K123 mutant forms of Tor2 [hereafter referred to as Tor2(K12) and Tor2(K123), respectively], Tor1-TORC1, Tor2-TORC1 and TORC2 complex-forming abilities were evaluated by co-immunoprecipitation (Fig. 4A,B). Analysis of Tor1-TORC1, in which HA-tagged Tor1 was pulled down together with Flag-tagged Kog1, showed that a higher amount of Tor1-TORC1 was detected in cells expressing Tor2(K12) than in cells expressing wild-type Tor2 (Fig. 4A, lane 2 versus lane 3). Analysis of Tor2-TORC1, in which HA-tagged Tor2 [either wild type Tor2 or the Tor2(K12) mutant] was pulled down together with Flag-tagged Kog1, indicated that the Tor2(K12) mutant had largely lost (34% of the wild type) its ability to form a Tor2-TORC1 complex with Kog1 (Fig. 4A, lane 4 versus lane 5). Nearly the same amount of Tor2-TORC1 was detected in the *tor1*Δ background as was found in the Tor1 wild-type strain (Fig. 4A, lane 4 versus lane 6). However, analysis of TORC2 using Flag-tagged Avo3 together with cell-based assays indicated that the Tor2(K12) mutant maintained TORC2-forming ability (70% of the wild type; Fig. 4B, lane 2 and 3). When the same amount of TORC2 was immunoprecipitated from cells expressing HA-tagged forms of either wild-type Tor2 or Tor2(K12) for *in vitro* TORC2 kinase assays, the TORC2 kinase activity was found to be similar, confirming that the mutation sites in Tor2(K12) did not affect the specific activity of TORC2 (Fig. 4C). A similar result was obtained by another method using ATPγS as a substrate, as also shown in Fig. 4C. These results show that the Tor2(K12) mutant was successfully designed as we expected; in cells expressing the Tor2(K12) mutant, Tor2-TORC1 formation is largely compromised, whereas TORC2 formation is well conserved. HA-tagged Tor2(K123) protein was barely detected in the cell lysate, although it was detected in denatured conditions (data not shown); therefore, we could not perform co-immunoprecipitation analysis for this mutant. The Tor2(K123) mutant complex is probably more fragile than the Tor2(K12) mutant complex, although it did form TORC2 and had TORC2 activity in cells. Therefore, for further experiments, we focused on the Tor2(K12) mutant and investigated functions of the two types of TORC1 by comparing *tor2*(K12) strains (where Tor2-TORC1 was almost lost in the cell) with the *tor1*Δ strain (where Tor1-TORC1 was lost).

### Phenotypes of *tor1*Δ and *tor2*(K12) mutant strains are different from each other

First, the *in vivo* TORC1 kinase activities in *tor1*Δ and *tor2*(K12) [*tor2*Δ expressing Tor2(K12) from a plasmid] strains were estimated by assaying the phosphorylation states of the TORC1 substrates Atg13 and Sch9 (Kamada et al., 2010; Urban et al., 2007). In both mutant strains, the activity of TORC1 was similar to that of the wild-type strain (Fig. 4D). Therefore, even if one of the two forms of TORC1 is lost, the TORC1 kinase activity against the major ligands is almost maintained. Note that the recovery rate of phosphorylation following re-addition of nutrients after starvation differs between the *tor1*Δ and *tor2*(K12) strains (Fig. S2A). Incidentally, TORC2 kinase activity against Mpk1 (also known as Slt2) (Fig. 4E) and actin organisation in cells (Kamada et al., 2005) (Fig. 4F) were also completely maintained in the *tor2*(K12) strain. Therefore, Tor2(K12)-TORC2 did not affect the phenotypes observed at 30°C. The *tor2*(K12) strain showed growth defects at 37°C (Fig. S2B).

**Fig. 5.**
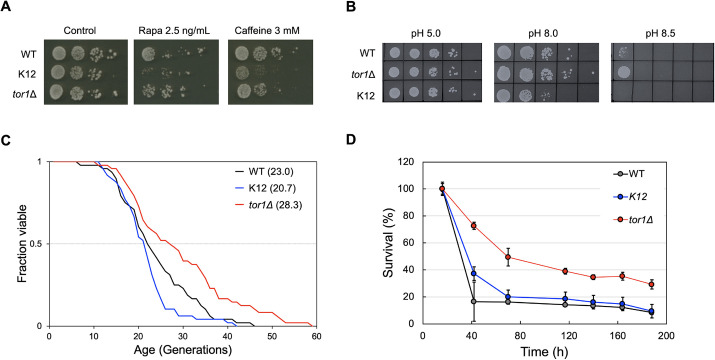
**Cell phenotypes induced by Tor2(K12).** (A) Cell growth in the presence of rapamycin (Rapa) or caffeine treatments. Growth of the *tor2*(K12) strain (K12) has higher sensitivity to 2.5 ng/ml rapamycin, a selective inhibitor of TORC1, and 3 mM caffeine, an inhibitor of TORC1, as compared to the wild-type (WT) and *tor1*Δ strains. The control data image is the same as that shown for 30°C data in Fig. S2B because these experiments were conducted at the same time with a shared control. (B) Cell growth at different pH conditions (pH 5.0, pH 8.0 and pH 8.5). The *tor1*Δ strain is viable at higher pH conditions, and the *tor2*(K12) strain is only viable at lower pH conditions, compared to the wild-type strain. Images in A and B are representative of 2–4 repeats. (C) Replicative lifespans of the wild-type (black), *tor2*(K12) (blue) and *tor1*Δ (red) strains. Mean life spans are shown in parentheses (*n*=48). Wilcoxon rank-sum test: *P*=0.030 (*tor1*Δ), *P*=0.20 (K12); weighted log-rank test: *P*=0.0054 (*tor1*Δ), *P*=0.065 (K12) (both versus the wild-type strain). (D) Chronological lifespans of the wild-type (black), *tor2*(K12) (blue) and *tor1*Δ (red) strains. Mean±s.d. (*n*=3). Loss of Tor1-TORC1 (*tor1*Δ strain) extends both replicative and chronological lifespans, whereas loss of Tor2-TORC1 [*tor2*(K12) strain] has a small effect on lifespan.

Next, we examined the cell phenotypes of the *tor2*(K12) and *tor1*Δ strains in the presence of TORC1 inhibitors (Fig. 5A; Fig. S2C). As previously reported, the *tor1*Δ strain is more sensitive than the wild-type strain to rapamycin, a selective inhibitor of TORC1, and caffeine, an inhibitor of TORC1 (Reinke et al., 2006; Sekiguchi et al., 2014). Interestingly, the phenotype of the *tor2*(K12) strain was distinct from that of the *tor1*Δ strain, with even greater sensitivity to rapamycin and caffeine than the *tor1*Δ strain. The sensitivity of the *tor1*Δ strain can be explained by a decrease in the total amount of TORC1. However, the hypersensitivity of the *tor2*(K12) strain cannot be explained by a decrease in TORC1, because the amount of Tor2-TORC1 is generally lower than that of Tor1-TORC1 (Loewith et al., 2002; Reinke et al., 2004). This result indicated that Tor2-TORC1 is distinct from Tor1-TORC1 in terms of its response to TORC1 inhibitors. Moreover, under several pH conditions, growth of the *tor2*(K12) and *tor1*Δ strains was observed (Fig. 5B). The *tor1*Δ strain grew better than the wild-type strain at high pH (pH 8.0 and pH 8.5). In contrast, *tor2*(K12) cells grew poorly at a high pH (pH 8.0) and did not grow at higher pH (pH 8.5). This result also indicates that Tor1-TORC1 has a role that is distinct from that of Tor2-TORC1.

Finally, replicative and chronological lifespans were measured for the wild-type, *tor1*Δ and *tor2*(K12) strains (Fig. 5C,D). Here, we used BY4741 genetic background strains, which are widely used for lifespan studies (Kaeberlein et al., 2005), and the *tor2*(K12) strain had the *tor2*(K12) allele integrated at the *TOR2* locus. The *tor1*Δ strain had a longer replicative lifespan than that of the wild-type strain, as previously reported (Kaeberlein et al., 2005). The replicative lifespan of the *tor2*(K12) strain was similar to that of the wild-type strain, and it also seemed slightly shorter. The mean lifespans of the wild-type, *tor1*Δ and *tor2*(K12) strains were 23.0, 28.3, and 20.7 generations, respectively. All strains had chronological lifespans similar to their replicative lifespans: the chronological lifespan of the *tor2*(K12) strain was similar to that of the wild-type strain, whereas the *tor1*Δ strain had a longer chronological lifespan than the wild-type strain. Both lifespan results suggest that the roles of Tor1-TORC1 and Tor2-TORC1 are different from each other. It is possible that the *tor2*(K12) strain is less affected because of the lower amount of Tor2-TORC1. However, since the TORC1 activity itself is almost the same in both the *tor2*(K12) and *tor1Δ* strains (Fig. 4D), it is more likely that Tor1-TORC1 and Tor2-TORC1 contribute in different ways to lifespan regulation. These phenotypic observations indicate that two types of TORC1 – Tor1-TORC1 and Tor2-TORC1 – not only have common and essential functions, but also have distinct functions.

## DISCUSSION

We engineered *S. cerevisiae* Tor2 based on computationally modelled Tor2-TORC1 and TORC2 structures. Through various cell biology and biochemical experiments, it was verified that the Tor2(K12) mutant maintains TORC2 activity but does not have TORC1 activity, as designed. Because only the TORC1-forming activity of Tor2 was disrupted, the *tor2*(K12) mutant strain provides a strain without Tor2-TORC1 function that was created without deletion of a gene, thus avoiding problems caused by both TORC1 and TORC2 being essential for cells. By comparing the phenotypes of the *tor2*(K12) mutant strain with those of the *tor1*Δ strain, we found that Tor2-TORC1 has functions that are distinct from those of Tor1-TORC1. Further research, for example, by solving and comparing high-resolution structures, could uncover the differences between Tor1-TORC1 and Tor2-TORC1 in detail.

In this study, we successfully altered the combinations of constituent proteins in protein complexes by using structure-based engineering, which has contributed to uncovering the biological functions of the protein complexes. Various proteins form complex states in cells, many of which alter the combination of constituent proteins to exert their functions at the correct place and time. Our approach, which artificially engineered a combination of constituent proteins, enabled us to control cellular phenotypes and uncover the roles of protein complexes. However, a reasonable structural model is essential for such structure-based engineering. In this study, we designed mutants based on model complex structures predicted computationally by homology modelling rather than experimental structures. Even if an experimental structure of a homologue protein is not available, AlphaFold2 (Jumper et al., 2021), a high-accuracy protein structure prediction program that uses deep learning, can be used to produce a predicted structure, and we can use high-quality structural data for any proteins that we are interested in engineering; note that experimental structures are better for designing mutants when they have been solved at high resolution. Therefore, our approach applies to any target protein, and we could engineer native proteins based on their structure and uncover their biological functions.

The phenotypic differences between the *tor2*(K12) and *tor1*Δ strains not only indicate that the role of Tor1-TORC1 is not the same as that of Tor2-TORC1 but also provide more detailed information about the characteristics of Tor1-TORC1 and Tor2-TORC1. The higher sensitivity of the *tor2*(K12) mutant strain to TORC1 inhibitors might indicate that Tor2-TORC1 has a lower binding affinity or a more robust response to the inhibitors than Tor1-TORC1, or that Tor2-TORC1 regulates different signal pathways to Tor1-TORC1; we will focus on these differences – for example, by measuring binding affinity for the purified protein samples – to reveal the detailed mechanisms in our future work. The pH dependence of cell growth might be related to the activation of V-ATPase, an ATP-driven proton pump, by TORC1. Transport across the plasma membrane by V-ATPase controls the cytoplasmic pH, and deletion of V-ATPase causes deceleration of growth at high pH conditions (Kane, 2006; Nelson and Nelson, 1990), suggesting that high V-ATPase activity might enable growth at high pH conditions. The activity of mammalian V-ATPase is dependent on the activation of mTORC1; the inactivation of mTORC1 regulates the formation of a complete and active complex state (Ratto et al., 2022). Therefore, it is expected that *S. cerevisiae* TORC1 activity also affects the activation of V-ATPase, and that TORC1 inactivation induces cell growth under alkali pH conditions. Our results were consistent with this expectation; the *tor1*Δ strain (permanent inactivation of Tor1-TORC1) grew under high pH conditions. In contrast, the *tor2*(K12) strain (almost permanently inactivated Tor2-TORC1) exhibited the opposite phenotype. These results should be investigated in more detail, and future research on these two strains might contribute to our understanding of the mechanism of V-ATPase activation by TORC1.

The findings obtained from this study could provide clues to the evolution of Tor2. The longer lifespan of the *tor1*Δ strain is the same phenotype as observed upon inhibition of mTORC1 or *S. pombe* TORC1, as previously reported (Harrison et al., 2009; Rodríguez-López et al., 2020). The pH dependence of growth of the *tor1*Δ strain (no Tor1-TORC1) is expected to be similar to the effects that would be induced by loss of mTORC1 function. Therefore, *S. cerevisiae* Tor1-TORC1 corresponds to TORC1 complexes from other species, as mentioned above. However, our results indicate that the roles of Tor2-TORC1 are not the same as those of Tor1-TORC1 in *S. cerevisiae* and TORC1 complexes in other species; they are characteristic of *S. cerevisiae* TORC1. This result also provides us information on the evolution of Tor kinases. A simple hypothesis is that *S. cerevisiae* Tor1-TORC1 is a special and later-emerging evolutionary adaptation because *S. cerevisiae* Tor2 forms both TORC1 and TORC2 complexes, similar to mTOR. However, our results indicate that Tor2-TORC1 has unique functions, and a simple hypothesis of the evolution process is not reasonable. By further investigation of Tor2-TORC1, we could better understand the evolution of Tor2.

We also obtained important findings regarding lifespan. Lifespan experiments showed that the *tor2*(K12) strain (which has almost no Tor2-TORC1) has a lifespan similar to that of the wild-type strain, whereas the *tor1*Δ strain (no Tor1-TORC1) has a longer lifespan. This result suggests that Tor1-TORC1 activity acts to shorten lifespan and that Tor2-TORC1 activity does not affect lifespan. By further investigating the differences between Tor1-TORC1 and Tor2-TORC1, we can uncover the function of *S. cerevisiae* Tor2-TORC1 in more detail. This knowledge might enable us to create an mTORC1 similar to *S. cerevisiae* Tor2-TORC1 and to extend the lifespan by replacing mTORC1 with a novel complex in mammals without inhibiting the activity of mTORC1. This will enable control of the lifespan of mammals.

## MATERIALS AND METHODS

### Design protocol for Tor2 mutants

The model structures of Tor2-TORC1 and TORC2 were created using following procedure. Homology models of Tor2, Kog1 and Avo3 were individually generated using the SWISS-MODEL server (Waterhouse et al., 2018). The model structures were superimposed on the mTORC1 (PDB ID: 6BCX) and mTORC2 (PDB ID: 6ZWM) cryo-EM structures. To remove clashes between the atoms of each component, the components were shifted slightly from each other and the side chains were repacked using the Rosetta protein design software (Leaver-Fay et al., 2011). These two model complex structures were compared, and candidate residues for mutations that contribute to binding to Kog1 in Tor2-TORC1 and not to Avo3 binding in TORC2 were selected. The hydrophobic residues of the candidate regions were mutated to a larger hydrophilic but uncharged amino acid, glutamine. Positively or negatively charged residues were mutated to larger amino acids with the same charge, for example, Lys to Arg. Several combinations of the candidate residues were experimentally validated.

### Strains, plasmids, media and genetic methods

The yeast strains, plasmids and DNA primers used in this study are listed in Tables S1, S2 and S3, respectively. The HA-TOR2 plasmid was provided by Dr J. Broach (Department of Molecular Biology, Princeton University, NJ, USA). Standard techniques were used to manipulate yeast (Kaiser et al., 1994; Longtine et al., 1998). YEPD and 5-FOA media were prepared using a previously reported protocol (Kaiser et al., 1994). Antibodies against HA epitope (16B12, COVANCE; 1:5000 dilution), Flag epitope (M2, Sigma; 1:5000 dilution), phospho-p44/42 (phospho-MAPK; 9101, Cell Signaling Technology; 1:3000 dilution), and thiophosphate ester-specific RabMAb recombinant monoclonal antibody (ab92570, Abcam; 1:5000 dilution) were used as primary antibodies for immunoblotting at the indicated dilutions. Antibodies against Atg13 (1:3000 dilution) were used as described previously (Kamada et al., 2000). The Flag-tagged Avo3 strain was generated following a previously described protocol (Longtine et al., 1998; Sung et al., 2005).

### Creation, cloning and cell-based assay of Tor2 mutants

Tor2 mutants were generated and cloned using the designated gap repair cloning (GRC) method (Chino et al., 2010; Ma et al., 1987). The overall procedure is presented in Fig. S3. First, mutated *TOR2* fragments were created by site-directed mutagenesis and amplified by PCR using specific DNA primers (Fig. S3A, Table S3). Next, these (two or three) DNA fragments were mixed and transformed into yeast cells with a linearised pRS314 vector (Fig. S3B). The plasmids created by GRC were rescued from yeast transformants.

The resultant *TOR2* mutant plasmids were transformed into *TOR1 tor2*Δ (YYK1411) and *tor1*Δ *tor2*Δ (YYK1412) strains harbouring the pRS316[TOR2] plasmid. The transformants were streaked onto 5-FOA plates to select *ura3^−^* cells that had lost the *URA3* marker plasmid carrying wild-type *TOR2* and harboured only the mutated *TOR2* plasmid. Growth on 5-FOA plates was used to evaluate the functions of TORC2 (*TOR1 tor2*Δ strain), and both TORC1 and TORC2 (*tor1*Δ *tor2*Δ strain). A YEPD plate was used as a growth control.

As for the integration of the *tor2*(K12) allele into the *TOR2* locus, the pRS314[TOR2(K12)] plasmid created above was cloned into BYP9689 (pBSBleMX), and the HindIII–HindIII (1.9 kb, encoding the FAT–FRB–kinase domains) region of the insert was deleted to make pBSBleMX[TOR2(K12) H3Δ]. The resulting plasmid was linearised by BamHI digestion and transformed into the BY4741 strain to generate the *tor2*::BleMX::*tor2*(K12) mutant. The phenotype of this strain was examined as shown in Fig. S4. This strain (YYK1551) was used for the lifespan assays.

### Immunoprecipitation of TORC1 and TORC2

Immunoprecipitation of the Tor complexes was performed as previously described (Kamada, 2017). For the TORC1 experiment, YYK1467 and YYK1530 (^HA^Tor2 ^Flag^Kog1 strains) were used, and for the TORC2 experiment, YYK1464 and YYK1528 (^HA^Tor2 Avo3^Flag^ strains) were used. Yeast cells grown in YEPD at 30°C overnight were collected and resuspended in Z buffer (50 mM Tris-HCl, pH 7.5, 1 M sorbitol) containing 0.01 mg/OD_600_ cells zymolyase 100T (Nacalai Tesque). The cells were converted to spheroplasts with 30 min incubation at 30°C. The spheroplasts were harvested, washed in Z buffer once, and suspended in 10 µl/OD_600_ cells ice-cold IP buffer [1× PBS, 2 mM MgCl_2_, 1 mM Na_3_VO_4_, 7.5 mM *p*-nitrophenyphosphate (*p*NPP), 10 mM β-mercaptoethanol, 1% Tween-20] containing protease inhibitors [40 µg/ml leupeptin, 80 µg/ml aprotinin, 20 µg/ml pepstatinA, 200 µg/ml 4-(2-aminoethyl) benzenesulfonyl fluoride hydrochloride (AEBSF) and 1 mM PMSF]. The cell suspension was gently mixed and incubated on ice for 5 min to break spheroplasts. The lysate was centrifuged 15,000 ***g*** at 4°C for 10 min twice, and the clear lysate (700 µl) was incubated with 15 µl of Dynabeads protein G (Invitrogen) bound with or without (control) 1 µl of anti-Flag antibody (M2, Sigma) 4°C for 2 h with gentle rotation. The beads were transferred into fresh microfuge tubes and washed thrice with 1× PBS or TES buffer (25 mM TES-KOH, pH 7.25, 100 mM KCl, 10 mM MgCl_2_). For immunoblotting, peroxidase-conjugated goat anti-rabbit IgG (H+L) or sheep anti-mouse IgG (H+L) (Jackson Immuno Research, West Grove, PA, USA) was used as a secondary antibody (1:10,000 dilution). Immobilon Forte Western HRP (Millipore) and Light-Capture II (ATTO, Tokyo, Japan) were used for signal detection (Kamada, 2017).

### *In vitro* TORC2 kinase assay

TORC2 kinase activity was evaluated using radioisotope (RI) and non-RI kinase assays as described previously (Allen et al., 2007; Kamada, 2017; Kamada et al., 2005). The resultant immunocomplex was washed once, suspended in 24 µl of TES buffer containing 2 µg of the substrate (^His6^4EBP1; Kamada et al., 2010), and preincubated at 30°C for 5 min. In the RI kinase assay, the reaction was initiated by the addition of 3 µl of 2 mM [γ-^32^P]ATP (222 TBq/mmol; Perkin Elmer) to the mixture (final concentration 0.2 mM; 0.2 MBq/reaction), and the reaction mixture (final volume 30 µl) was further incubated at 30°C for 10 min. The reaction was terminated by adding 15 µl 4× SDS-PAGE sample buffer (Sambrook et al., 1989) and incubating at 65°C for 5 min. The samples (20 µl) were subjected to SDS-PAGE (12.5%), and the phosphorylated proteins were analysed using autoradiography and a BAS5000 (Fuji Film). In the non-RI assay, the reaction was initiated by adding 6 µl of 1 mM ATPγS (Abcam) to the mixture (final concentration 0.2 mM), and the reaction mixture (final volume 30 µl) was further incubated at 30°C for 20 min. The reaction was terminated by adding 3 µl of 250 mM EDTA. The protein in the reaction mixture was alkylated with 1.7 µl of 50 mM *p*-nitrobenzyl mesylate (*p*NBM; 2.7 mM, Abcam) at room temperature for 80 min. The sample was added to 12 µl of 4× SDS-PAGE sample buffer and incubated at 95°C for 2 min, and a 20 µl aliquot was subjected to SDS-PAGE (12.5%). The phosphorylated substrate was analysed by immunoblotting as described above using thiophosphate ester-specific RabMAb (Abcam) as the primary antibody. The substrate protein (4EBP1) was visualised using Coomassie Brilliant Blue (CBB; GelCode Blue Protein Stain, Thermo Fisher Scientific) or Ponceau S [Ponceau; 0.1% Ponceau S (Sigma) in 5% acetic acid].

### *In vivo* kinase analyses

Immunoblotting was performed as previously described (Kamada, 2017). Yeast cells harbouring YEp352[ATG13], YCplac33[^HA^SCH9] or YEp352[MPK1^HA^] (Kamada et al., 1995) grown in YEPD medium at 30°C. For the nitrogen starvation treatment, cells were collected, washed thrice with distilled water, transferred to synthetic dextrose (SD) (−N) medium (0.17% yeast nitrogen base without ammonium sulfate and amino acids, 2% glucose), and incubated for 30 min. Cells (10 OD_600_ units) were collected and fixed with 100 µl of ice-cold alkaline solution (0.2 N NaOH and 0.5% β-mercaptoethanol). After 5 min of incubation on ice, 10 µl of 1.8 M sodium acetate pH5.2 and 1 ml of ice-cold acetone were added to the sample and incubated at −20°C to precipitate the proteins. The protein samples were precipitated with a microfuge for 5 min (15,000 ***g***), air-dried, suspended in 100 µl of SDS-PAGE sample buffer, and incubated at 65°C for 15 min. The samples were thoroughly dissolved by sonication and subjected to SDS-PAGE. Immunoblotting was performed as described above.

### Actin staining

Staining for actin was performed and observed as described previously (Kamada et al., 2005; Nakano et al., 2011). YEPD-grown cells were fixed for 30 min by the direct addition of 37% formaldehyde stock to a final concentration of 5%. Fixed cells were collected, washed with 1× PBS thrice, and then BODIPY–phallacidin (Molecular Probes) was added for 2 h at room temperature to stain F-actin as previously described (Kaiser et al., 1994). Fluorescence and bright-field images were captured using a Personal DV microscope (Applied Precision). Fluorescence images were acquired in 20 serial sections along the *z*-axis at intervals of 0.2 mm. All images were three-dimensionally deconvolved and stacked using the quick projection algorithm in the SoftWoRx software (Applied Precisions).

### Replicative lifespan assay

The replicative lifespan was measured for the wild-type (BY4741), *tor1*Δ (YYK332) and *tor2*(K12) (YYK1551) strains. Replicative lifespan was assayed as previously described (Nakajima et al., 2020). Yeast cells were thawed from the frozen stock and streaked onto YEPD agar plates. After 2 days, a single colony was spread onto a YEPD agar plate, and the cells were grown at 30°C overnight. The next day, cells were transferred again to fresh YEPD agar plates and grown overnight. Using a micromanipulator, 48 cells were arrayed on a plate and allowed to undergo one or two divisions. Virgin cells were then selected and subjected to lifespan analysis. Except during manipulation, the plates were sealed with Parafilm, incubated at 30°C during the day and stored at 4°C at night to avoid excessive budding. Daughter cells were removed by gentle agitation using a dissecting needle and scored every 2 h. For each of the 48 cell lines, buds from each mother cell were counted for at least 3 days until the division of living cells ceased. The mean replicative lifespan and the *p*-value were calculated using the Wilcoxon rank-sum test and weighted log-rank test relative to the wild-type strain, BY4741.

### Chronological lifespan assay

The same cell strains as those used in the replicative lifespan assay were used. To measure cell survival, cells were grown in SD liquid medium, sampled during each growth phase, and plated on yeast extract agar plates after dilution (Ohtsuka et al., 2021a). After 4–7 days at 30°C, using colony-forming units, the number of viable cells in 1 ml aliquots of culture was determined and divided based on the cell turbidity at each sampling time. Cell growth was then monitored according to the turbidity determined using a Bactomonitor (BACT-550) equipped with a 600 nm filter (Nissho Electric). To measure the chronological lifespan, the wild-type (BY4741), *tor1*Δ (YYK332) and *tor2*(K12) (YYK1551) strains were cultured in SD medium with 240 mg/l leucine, 80 mg/l uracil, 80 mg/l histidine, and 80 mg/l methionine (*n*=3).

## Supplementary Material



10.1242/joces.261625_sup1 Supplementary information
