# Supplementary material for: Structure-based engineering of Tor complexes reveals that two types of yeast TORC1 produce distinct phenotypes
Source: J Cell Sci. 2024 Feb 28;137(4):jcs261625. doi: 10.1242/jcs.261625 (PMC10941655; doi:10.1242/jcs.261625)
Supplement: Supplementary information [file joces-137-261625-s1.pdf]

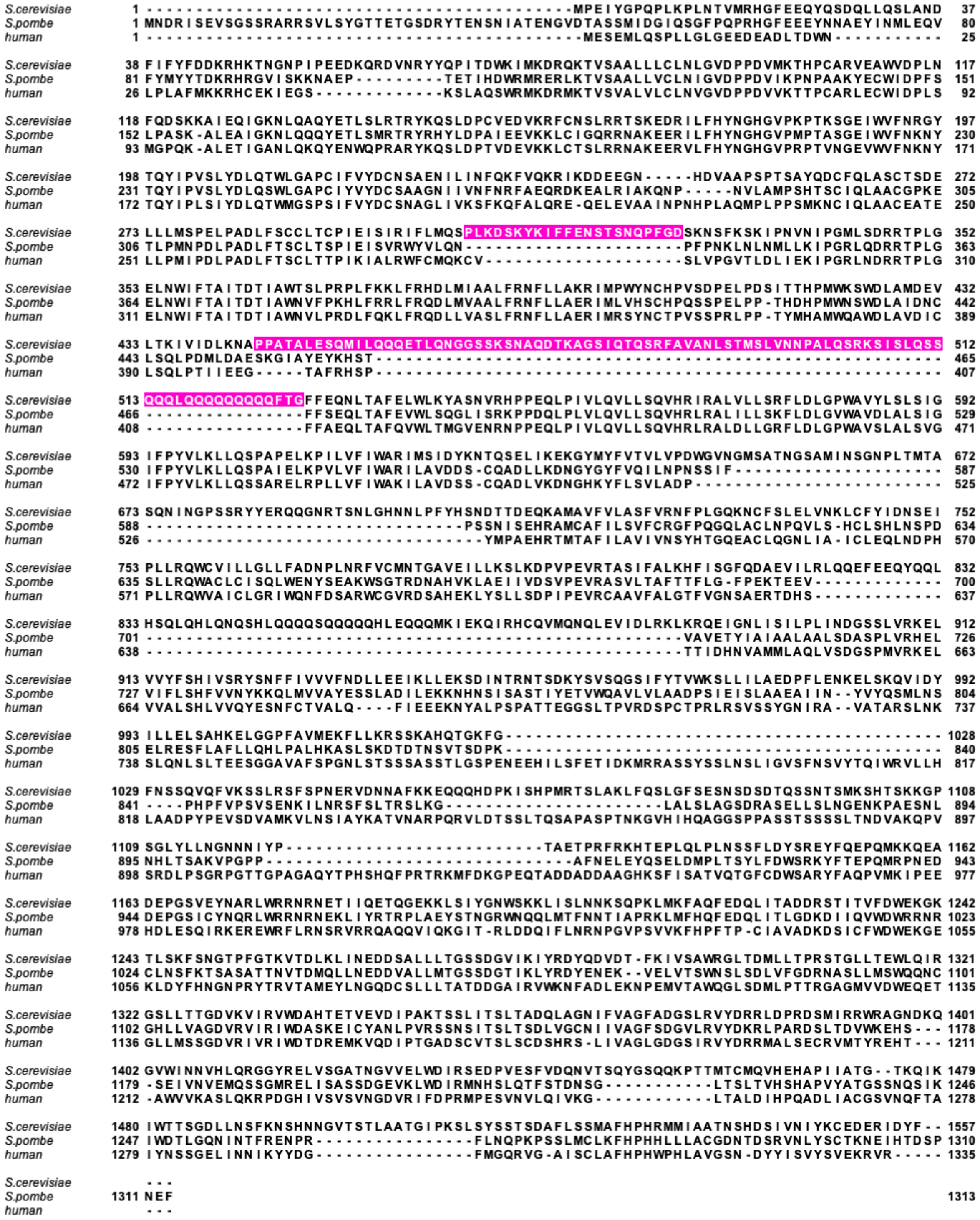


**Fig. S1. Sequence alignment among Kog1 and the orthologs.**

Only Kog1 from *S. cerevisiae* has insertions (Pink); the insertion loops interact with Tor2 in our model structures.

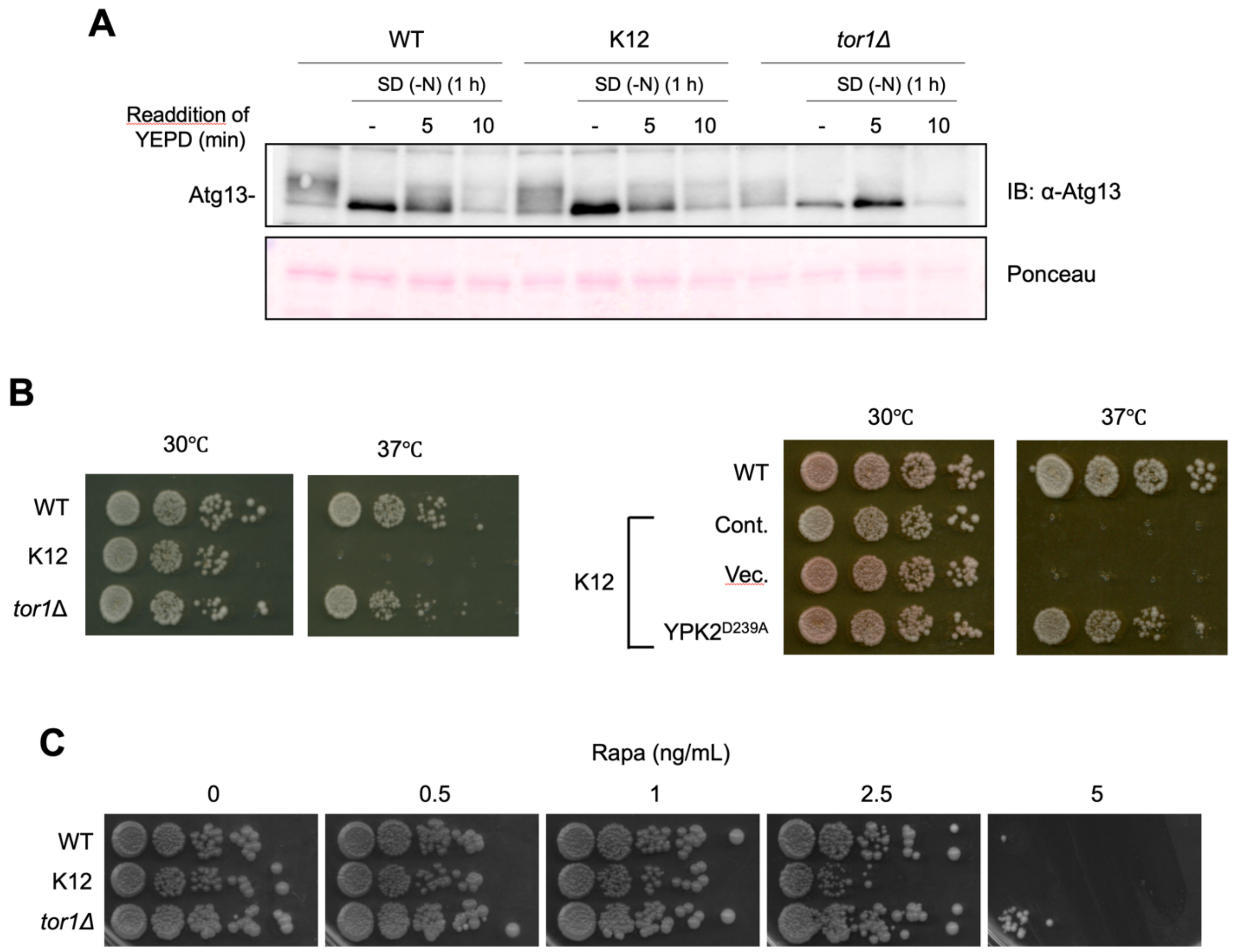


**Fig. S2. Additional characteristics of Tor2(K12) mutant.**

**A.** Recovery of Atg13 phosphorylation by re-addition of nutrient after starvation. The wildtype, *tor2*(K12), and *tor1Δ* strains harbouring ATG13 plasmid growing in YEPD at 30 °C were treated by nitrogen starvation (-N) for 1 hour. The phosphorylation states of Atg13 were examined by immunoblotting at 0, 5, and 10 min after re-addition of nutrient. The wildtype and *tor2*(K12) strains were recovered quickly. The recovery of *tor1Δ* strain was slower than others.

**B.** Cell growth at high temperature (37°C). Left: Cell growth of the wildtype, *tor2*(K12) and *tor1Δ* strains at 37°C. *tor2*(K12) strain is sensitive at higher temperature (37 °C). The figure of 30 °C data is the same as that of control data in Fig. 5A, because these experiments were conducted at the same time with a shared control. Right: Cell growth of *tor2*(K12) strain with YPK2 mutant D239A). YPK2$^{D239A}$ mutant has been reported to constantly activate TORC2 signal pathway(Kamada et al., 2005). By the mutation of YPK2, temperature sensitivity of *tor2*(K12) strain was suppressed. This result indicates the temperature sensitivity is caused by decreased TORC2 activity of *tor2*(K12) strain.

**C.** Cell growth under different dilution series of rapamycin (Rapa). Cell growth of the wildtype, *tor2*(K12) and *tor1Δ* strains were observed under 0, 0.5, 1, 2.5, and 5 g/mL rapamycin. *tor2*(K12) strain is more sensitive to rapamycin than *tor1Δ* strain.

Journal of Cell Science • Supplementary information

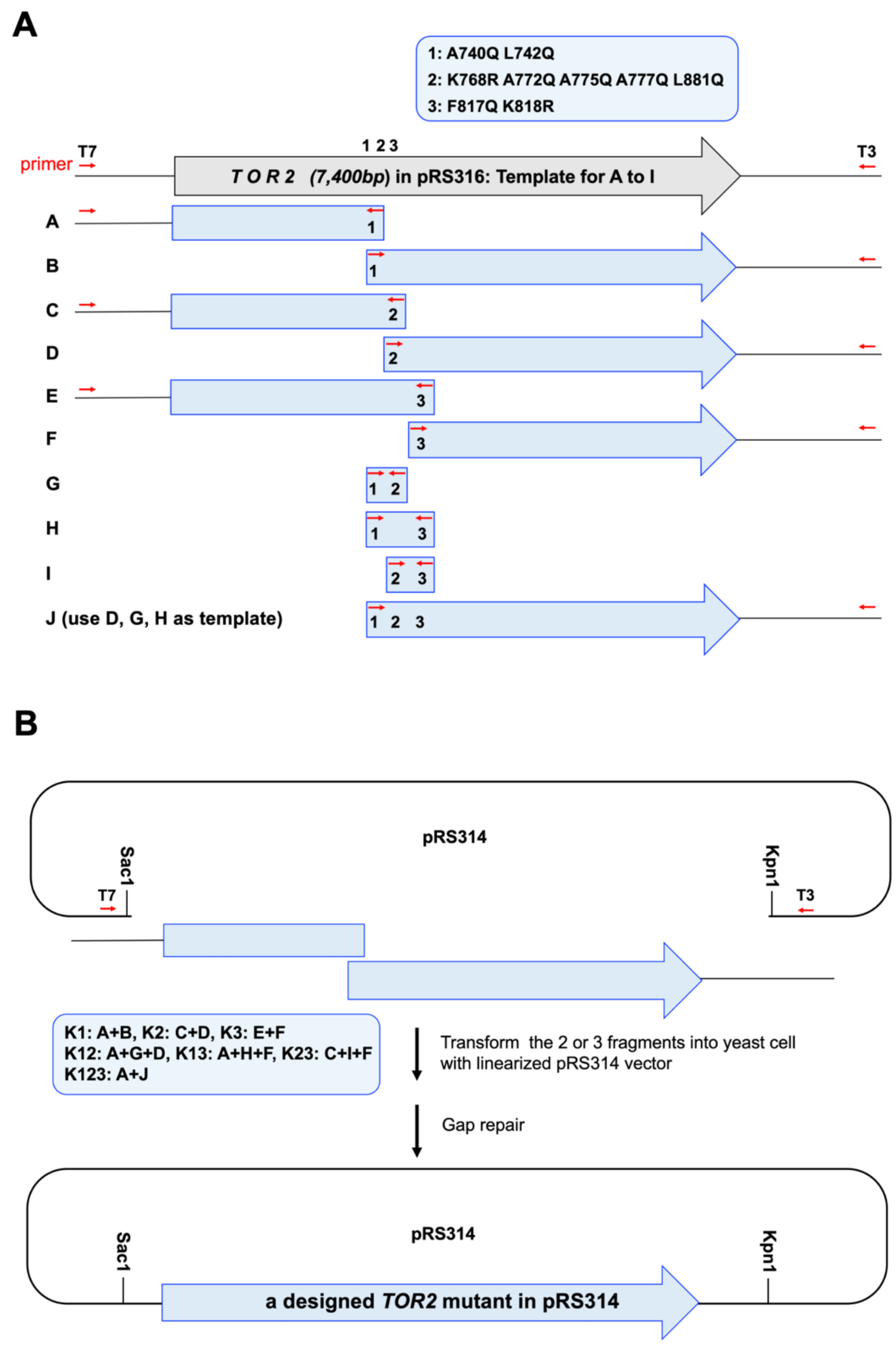


**Fig. S3. Protocol to construct Tor2 mutant plasmids.**

**A.** gene fragments of designed Tor2 mutants were amplified by PCR using DNA primers.

**B.** the gene fragments were mixed with linearized pRS314 vector and transformed into yeast cells. The plasmids of designed Tor2 were extracted from the transformed yeast cells.

Journal of Cell Science • Supplementary information

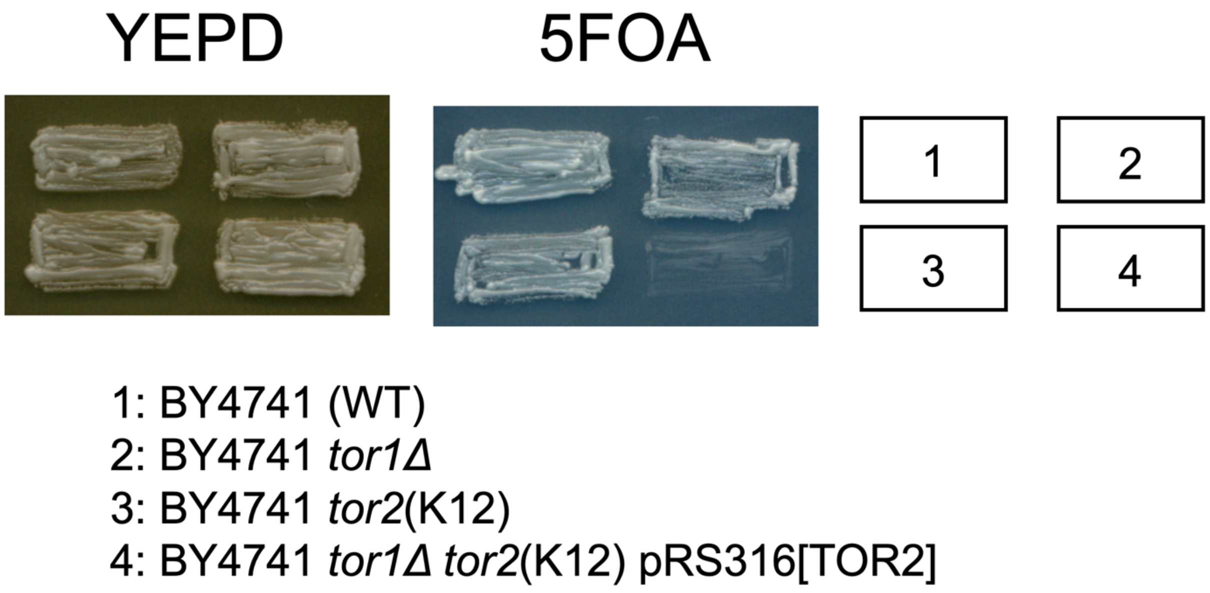


**Fig. S4. Cell-based assay of *tor2*(K12) strain derived from BY4741.**

Cell-based activity assay indicated *tor2*(K12) strain derived from the BY4741 also have no TORC1 activity and keep TORC2 activity, as is the case with that from the W303 (Fig. 3C).

Journal of Cell Science • Supplementary information

**Table S1. Yeast strains used in this study.**

| *Strain* | *Genotype* | *Source* |
|---|---|---|
| W303a | *Mat**a**ade2 his3 leu2 trp1 ura3 can1* | Laboratory stock |
| W303α | *Mat**α** ade2 his3 leu2 trp1 ura3 can1* | Laboratory stock |
| BY4741 | *Mat**a** his3Δ1 leu2Δ0 met15Δ0 ura3Δ1* | Laboratory stock |
| YYK332 | BY4741 *tor1*Δ::KanMX | Laboratory stock |
| YYK799 | W303α *tor1*Δ:: KanMX pRS313[$^{HA}$TOR1] pRS316[$^{Flag}$KOG1] | This study |
| YYK1411 | W303α *tor2*Δ::*HIS3* pRS316[TOR2] | This study |
| YYK1412 | W303a *tor1*Δ::KanMX *tor2*Δ::HIS3 pRS316[TOR2] | This study |
| YYK1414 | W303α *tor2*Δ::*HIS3* pRS314[TOR2$^{WT}$] | This study |
| YYK1418 | W303a *tor1*Δ::KanMX *tor2*Δ::HIS3 pRS314[TOR2$^{WT}$] | This study |
| YYK1448 | W303α *tor2*Δ::*HIS3* AVO3$^{Flag}$::HphMX pRS316[TOR2] | This study |
| YYK1449 | W303a *tor1*Δ::KanMX *tor2*Δ::HIS3 AVO3$^{Flag}$::HphMX pRS316[TOR2] | This study |
| YYK1464 | W303α *tor2*Δ::*HIS3* AVO3$^{Flag}$::HphMX pRS314[$^{HA}$TOR2$^{WT}$] | This study |
| YYK1465 | W303α *tor2*Δ::*HIS3* AVO3$^{Flag}$::HphMX pRS314[$^{HA}$TOR2 K123] | This study |
| YYK1467 | W303α *tor2*Δ::*HIS3* pRS314[$^{HA}$TOR2$^{WT}$] pRS316[$^{Flag}$KOG1] | This study |
| YYK1468 | W303α *tor2*Δ::*HIS3* pRS314[$^{HA}$TOR2 K123] pRS316[$^{Flag}$KOG1] | This study |
| YYK1470 | W303a *tor1*Δ::KanMX *tor2*Δ::HIS3 pRS314[$^{HA}$TOR2$^{WT}$] pRS316[$^{Flag}$KOG1] | This study |
| YYK1499 | W303α *tor2*Δ::*HIS3* pRS314[TOR2 K12] | This study |
| YYK1500 | W303α *tor2*Δ::*HIS3* pRS314[TOR2 K13] | This study |
| YYK1501 | W303α *tor2*Δ::*HIS3* pRS314[TOR2 K23] | This study |
| YYK1502 | W303a *tor1*Δ::KanMX *tor2*Δ::HIS3 pRS314[TOR2 K13] | This study |
| YYK1503 | W303a *tor1*Δ::KanMX *tor2*Δ::HIS3 pRS314[TOR2 K23] | This study |
| YYK1528 | W303α *tor2*Δ::*HIS3* AVO3$^{Flag}$::HphMX pRS314[$^{HA}$TOR2 K12] | This study |
| YYK1530 | W303α *tor2*Δ::*HIS3* pRS314[$^{HA}$TOR2 K12] pRS316[$^{Flag}$KOG1] | This study |
| YYK1551 | BY4741 *tor2*::BleMX::*TOR2* K12 | This study |
| YYK1553 | BY4741 *tor1*Δ::NatMX *tor2*::BleMX::*TOR2* K12 | This study |
| YYK1580 | W303a *tor1*Δ::KanMX *tor2*Δ::HIS3 pRS315[$^{HA}$TOR1] pRS314[TOR2 K12] pRS316[$^{Flag}$KOG1] | This study |

Journal of Cell Science • Supplementary information

**Table S2. Plasmids used in this study.**

| *Name* | *Source* |
|---|---|
| pRS316[TOR2] | Laboratory stock |
| pRS314[TOR2] | Laboratory stock |
| pRS314[TOR2 K1] | This study |
| pRS314[TOR2 K2] | This study |
| pRS314[TOR2 K3] | This study |
| pRS314[TOR2 K12] | This study |
| pRS314[TOR2 K13] | This study |
| pRS314[TOR2 K23] | This study |
| pRS314[TOR2 K123] | This study |
| pRS314[$^{HA}$TOR2] | Laboratory stock |
| pRS314[$^{HA}$TOR2 K12] | This study |
| pRS314[$^{HA}$TOR2 K123] | This study |
| pRS313[$^{HA}$TOR1] | Laboratory stock |
| pRS315[$^{HA}$TOR1] | Laboratory stock |
| pRS316[$^{Flag}$KOG1] | Laboratory stock |
| YEp352[*ATG13*] | Laboratory stock |
| YCplac33[$^{HA}$*SCH9*] | Laboratory stock |
| YEp352[MPK1$^{HA}$] | Laboratory stock |
| YEp352[YPK2$^{D239A}$] | Kamada et al. 2005 |
| BYP9689 | NBRP Yeast |
| BYP9689[TOR2(K12) H3Δ] | This study |

Journal of Cell Science • Supplementary information

**Table S3. DNA primers used in this study.**

| *Name* | *Sequence* |
|---|---|
| AVO3-F2 | GGGACAAAAGGCCGGCTAATTTTACACGTCGGATCCCCGGGTTAATTAA |
| AVO3-R1rc | CTATATACATTTATACATGCGGCCCTTTTTTGCTGAATTCGAGCTCG |
| AVO3-4991 | CAATACCGCAATGACGATGACTCCATCG |
| AVO3-5935rc | GTAAGTCACGTGAAATATTTCCTGTGGC |
| TOR2seq-3104 | GGCAGATTGAGTTCTGTCAACCCCG |
| TOR2-961 | GGCAGATTGAGTTCTGTCAACCCCG |
| TOR2-3578rc | CAAACGAACAGTTCCACGCCTGATATGAGG |
| T3-PCR | CCAAGCGCGCAATTAACCCTCACTAAAGGG |
| T7-PCR | TGAGCGCGCGTAATACGACTCACTATAGGG |
| TOR2seq701 | GGGCGATGTCAGAATATTTACCCGG |
| TOR2seq1200 | GTCATATTGGTAAAATATCCTTTGT |
| TOR2seq1701 | CTAAGTTGGAATACAGGAGACATGC |
| TOR2seq2203 | GAAGAACATCGATATGAATGCTGCA |
| TOR2seq2706 | CAGATGCCCAAATTTTGATTCAGTG |
| TOR2seq3208 | GGAGGAAAGTGCAACTCTATTATGT |
| TOR2seq3703 | CGACATCGCATTGCTAATGCAAGGG |
| TOR2seq4206 | CTTTGGTTACTTTTGGGCCGAATCT |
| TOR2seq4702 | GAAGACCAAAGAAGATTGGCAAGAA |
| TOR2seq5203 | GGAAACTTGGTATGAAAAACTTCAA |
| TOR2seq5702 | GATAAACGTCTAACTATGAGAGAAA |
| TOR2seq6201 | GATTGAGCAATCCAGATTCGATCCT |
| TOR2seq6705 | CTGATCTAGGTAAGGCTCATCCGCA |

Journal of Cell Science • Supplementary information

| *Name* | *Sequence* |
|---|---|
| TOR2seq7201 | GCCAAAACTACTATCTGCGCATGAT |
| TOR2seq7704 | CGGAGGGACAAGATCTTTATAAGGT |
| TOR2seq8205 | CCGAAGAAGAAGTTCAAAGGGTGGA |
| TOR2L527Q-1 | TTACTGTCGATATCCCAATCTGGTGAAAAA |
| TOR2L527Q-1rc | TTTTTCACCAGATTGGGATATCGACAGTAA |
| TOR2I568Q-2 | GAATCTAATGACGATCAAACAGATGCCCAA |
| TOR2I568Q-2rc | TTGGGCATCTGTTTGATCGTCATTAGATTC |
| TOR2D606EK611R-3 | ACATTGAGCATGAGGAGTCGTCTGTCAGACGTCTGGCAGCATTAACG |
| TOR2D606EK611R-3rc | CGTTAATGCTGCCAGACGTCTGACAGACGACTCCTCATGCTCAATGT |
| TOR2A740QL742Q-4 | CAAAAAAAAAGGAGGAAAGTCAAACTCAGCTGTGTACGCTGATAAATTC |
| TOR2A740QL742Q-4rc | GAATTTATCAGCGTACACAGCTGAGTTTGACTTTCCTCCTTTTTTTTTG |
| TOR2K768RtoL781Q-5 | GACGTCATTCTTCCGCGGTGCCAGGATCAATCATCTCAAGTACAATCCACCGCTCAAAAGGTTTTGGGTGAAC |
| TOR2K768RtoL781Q-5rc | GTTCACCCAAAACCTTTTGAGCGGTGGATTGTACTTGAGATGATTGATCCTGGCACCGCGGAAGAATGACGTC |
| TOR2F817QK818R-6 | GACCAATCAAACTCTCAAAGAAGAGATGCCGCCTTAAC |
| TOR2F817QK818R-6rc | GTTAAGGCGGCATCTCTTCTTTGAGAGTTTGATTGGTC |
| TOR2 922 | CATAAAGAGCATAGICATTAAGATCAAATAGTTATC |
| TOR2 8529rc | GTTAGTAACGTCACGCTCGGAACTAAACATTAAIG |

Journal of Cell Science • Supplementary information
