# Supplementary material for: Structure-based engineering of Tor complexes reveals that two types of yeast TORC1 produce distinct phenotypes
Source: J Cell Sci. 2024 Feb 28;137(4):jcs261625. doi: 10.1242/jcs.261625 (PMC10941655; doi:10.1242/jcs.261625)
Supplement: Supplementary file 1 [file joces-137-261625_review_history.pdf]

Structure-based engineering of Tor complexes reveals that two types of yeast TORC1 produce distinct phenotypes

Yoshiaki Kamada, Chiharu Umeda, Yukio Mukai, Hokuto Ohtsuka, Yoko Otsubo, Akira Yamashita and Takahiro Kosugi

DOI: 10.1242/jcs.261625

Editor: Tamotsu Yoshimori

Review timeline

Original submission:	6 September 2023
Editorial decision:	26 September 2023
First revision received:	29 December 2023
Accepted:	18 January 2024

Original submission

First decision letter

MS ID#: JOCES/2023/261625

MS TITLE: Structure-based engineering of Tor complexes uncovers different roles of two types of yeast TORC1s

AUTHORS: Yoshiaki Kamada, Chiharu Umeda, Yukio Mukai, Hokuto Ohtsuka, Yoko Otsubo, Akira Yamashita, and Takahiro Kosugi

ARTICLE TYPE: Research Article

We have now reached a decision on the above manuscript.

To see the reviewers' reports and a copy of this decision letter, please go to: https://submit-jcs.biologists.org and click on the 'Manuscripts with Decisions' queue in the Author Area. (Corresponding author only has access to reviews.)

As you will see, the reviewers are positive but raise several substantial criticisms that prevent me from accepting the paper at this stage. They suggest, however, that a revised version might prove acceptable, if you can address their concerns. If you think that you can deal satisfactorily with the criticisms on revision, I would be pleased to see a revised manuscript. We would then return it to the reviewers.

Please ensure that you clearly highlight all changes made in the revised manuscript. Please avoid using 'Tracked changes' in Word files as these are lost in PDF conversion.

I should be grateful if you would also provide a point-by-point response detailing how you have dealt with the points raised by the reviewers in the 'Response to Reviewers' box. Please attend to all of the reviewers' comments. If you do not agree with any of their criticisms or suggestions please explain clearly why this is so.

Reviewer 1

© 2024. Published by The Company of Biologists under the terms of the Creative Commons Attribution License (https://creativecommons.org/licenses/by/4.0/).

Advance summary and potential significance to field

Target of rapamycin (Tor) is an evolutionarily conserved protein kinase that senses nutritional states of the cell. It is known that Tor functions by forming two distinct complexes, Tor complex 1 (TORC1) and complex 2 (TORC2). In Saccharomyces cerevisiae, Tor1 constitutes TORC1 whereas Tor2 constitutes both TORC1 and TORC2. As a result, there exist two types of TORC1: Tor1-containing TORC1 (Tor1-TORC1) and Tor2-containing TORC1 (Tor2-TORC1). Thus far, the different roles of TORC1 and TORC2 have been studied well, it remained elusive whether Tor1-TORC1 and Tor2-TORC1 play different roles. In this manuscript, the authors performed homology modeling of S. cerevisiae Tor2-TORC1 and TORC2 structures and designed Tor2 mutant that lost the ability to constitute TORC1 but retained the ability to constitute TORC2. Moreover, analyses using the Tor2 mutant revealed for the first time that Tor1-TORC1 and Tor2-TORC1 induced different phenotypes in rapamycin, caffeine and pH dependences of cell growth and replicative and chronological lifespans.
The fact that the authors succeeded in obtaining strains containing only each of the similar complexes (Tor1-TORC1 and Tor2-TORC1) in a very well thought-out manner and revealing the differences in function for the first time is highly commendable, especially with regard to the comparison of the functions of similar complexes that are usually abandoned in analyses. The fact that the same method can be used to solve similar problems in other systems is also very useful. It is also commendable that the strategies made in this paper can be applied to other systems with similar problems. Therefore, this manuscript will be of interest to a wide range of researchers in cell biology. There are several concerns with the data listed below, which need to be addressed prior to be published at this journal.

Comments for the author

1) The authors compared the phenotypes of tor1-delta strain (containing Tor2-TORC1) and tor2(K12 mutant) strain (containing Tor1-TORC1). However, they did not compare the amount of Tor2-TORC1 and Tor1-TORC1 and so the different phenotypes might be due to the difference in their amount. Compare the amount of Tor2-TORC1 and Tor1-TORC1 and if a large difference is observed (possibly the amount of Tor1-TORC1 would be larger than that of Tor2-TORC1), discuss the effect of the amount difference in the different phenotypes.

2) The authors wrote that “Tor2(K12) mutant largely loses its ability to form a Tor2-TORC1 complex with Kog1” and that “the K12 mutant maintained sufficient Tor2-TORC2 forming ability to function as TORC2” based on the data of Figure 4A, B.
However, the data show that K12 appears to have retained a bit of affinity with Kog1 and also lost a bit of affinity with Avo3. Repeat the experiments and quantify the data and discuss the impact of slightly remaining interactions and slightly decreasing interactions on the phenotypes.

3) Provide molecular weight marker information for the blotting data of Figure 4.

4) At line 75, “the function of Tor2-TORC1 function”, delete the second “function”.

<u>Reviewer 2</u>

Advance summary and potential significance to field

This manuscript entitled “Structure-based engineering of Tor complexes uncovers different roles of two types of yeast TORC1s” by Kamada et al. uncovered difference of Tor1 and Tor2 in TORC1 complex. Based on computer modelling, they introduce the mutations in Tor2 that prevent the assemble with TORC1 bun not with TORC2. By using these mutants, they showed that the cell with TORC1 either only containing TOR1 or TOR2 showed different phenotypes in rapamycin sensitivity and replicative and chronological lifespans. These suggest TOR1 and TOR2 provide different contribution in TORC1 function.

This is interesting study based on cutting edge protein engineering regarding multiprotein complex. In spite of crucial role of TORC1 in yeast, the difference between Tor1 and Tor2 had been

© 2024. Published by The Company of Biologists under the terms of the Creative Commons Attribution License (https://creativecommons.org/licenses/by/4.0/).

overlooked. This study succeeded in dissection of the difference using protein engineering. Basically, the analysis was clearly conveyed, but some critical controls are missing.

Comments for the author

1. Fig 4A. Which band is Flag-Kog1 especially in Total lysate? Negative control without expression of Flag-Kog1 is needed. It is not appropriate to cut the blot nearly at the bottom band in IP. Is the bottom band Kog1?

2. Fig4B. Likewise, which is the Avo3-Flag band in Total lysate? Negative control is needed.

3. Fig 4D. There seem to exist subtle difference in Atg13 and Sch9 phosphorylation in tor1D cells. It is possible as Tor1 is a major component in TORC1 than Tor2. If it is true, it will affect the interpretation of in vivo data. More detailed evaluation is required in this figure. For example, how about the activation by re-addition of nutrient after starvation?

4. Fig 4E Negative control in which TORC2 activity is suppressed is required to evaluate the degree of phosphorylation.

5. Fig 4F. Again, negative control in which TORC2 activity is suppressed and actin organization is perturbed is required. Otherwise, readers cannot evaluate these images.

6. Fig 5A. The result that K12 is more sensitive to rapamycin than tor1D is interesting and important in this study. This data should be presented in more comprehensive manner using different dilution series of rapamycin to clearly show the difference.
Furthermore, more detailed is required regarding discussion rapamycin mediated inhibition and the structure modified in this study.

7. 'Different roles' in title is over interpreted. Some tone down is required.

First revision

Author response to reviewers' comments

We uploaded this Response as Supplementary Information (Response Letter).

Reviewer comments:

Reviewer 1 Advance Summary and Potential Significance to Field:

Target of rapamycin (Tor) is an evolutionarily conserved protein kinase that senses nutritional states of the cell. It is known that Tor functions by forming two distinct complexes, Tor complex 1 (TORC1) and complex 2 (TORC2). In Saccharomyces cerevisiae, Tor1 constitutes TORC1 whereas Tor2 constitutes both TORC1 and TORC2. As a result, there exist two types of TORC1: Tor1-containing TORC1 (Tor1-TORC1) and Tor2-containing TORC1 (Tor2-TORC1). Thus far, the different roles of TORC1 and TORC2 have been studied well, it remained elusive whether Tor1-TORC1 and Tor2-TORC1 play different roles. In this manuscript, the authors performed homology modeling of S. cerevisiae Tor2- TORC1 and TORC2 structures and designed Tor2 mutant that lost the ability to constitute TORC1 but retained the ability to constitute TORC2. Moreover, analyses using the Tor2 mutant revealed for the first time that Tor1-TORC1 and Tor2-TORC1 induced different phenotypes in rapamycin, caffeine and pH dependences of cell growth and replicative and chronological lifespans.
The fact that the authors succeeded in obtaining strains containing only each of the similar complexes (Tor1-TORC1 and Tor2-TORC1) in a very well thought-out manner and revealing the differences in function for the first time is highly commendable, especially with regard to the comparison of the functions of similar complexes that are usually abandoned in analyses. The fact that the same method can be used to solve similar problems in other systems is also

© 2024. Published by The Company of Biologists under the terms of the Creative Commons Attribution License (https://creativecommons.org/licenses/by/4.0/).

very useful. It is also commendable that the strategies made in this paper can be applied to other systems with similar problems. Therefore, this manuscript will be of interest to a wide range of researchers in cell biology. There are several concerns with the data listed below, which need to be addressed prior to be published at this journal.

We sincerely appreciate your comments. We improved our manuscript according to your suggestions. Line-by-line responses are described in the following.

Reviewer 1 Comments for the Author:

1) The authors compared the phenotypes of tor1-delta strain (containing Tor2-TORC1) and tor2(K12 mutant) strain (containing Tor1-TORC1). However, they did not compare the amount of Tor2-TORC1 and Tor1-TORC1 and so the different phenotypes might be due to the difference in their amount. Compare the amount of Tor2-TORC1 and Tor1- TORC1 and if a large difference is observed (possibly the amount of Tor1-TORC1 would be larger than that of Tor2-TORC1), discuss the effect of the amount difference in the different phenotypes.

We compared the amount of Tor2-TORC1 in the *tor1Δ* strain and Tor1-TORC1 in the *tor2*(K12) strain, and also compared the total amount of TORC1 among the strains, the wild-type, *tor2*(K12), and *tor1Δ* strains, because it would be better to discuss the effect of amount based on the total amounts if two types of TORC1 have the same functions. The HA-tagged Tor1 or Tor2 was pulled down with the Flag-tagged Kog1 for the wild-type, *tor2*(K12), and *tor1Δ* strains (revised Figure 4A). The amount of Tor2-TORC1 in the *tor1Δ* strain was similar to that in the wildtype (lane 4and 7 in the revised Figure 4A). On the other hand, interestingly, the amount of Tor1-TORC1 in the *tor2*(K12) strain was higher than that in the wildtype (lane 2 and 4 in the revised Figure 4A). In addition, the Tor2-TORC1 in the wildtype was detected without much difference from Tor1-TORC1 in the wildtype; the amount of Tor2-TORC1 seems to be slightly less than that of Tor1-TORC1 (lane 2 and 4 in the revised Figure 4A). Summarizing the total amount of TORC1 (sum of Tor1-TORC1 and Tor2-TORC1) for the wild-type, *tor2*(K12), and *tor1Δ* strains, the wildtype and *tor2*(K12) strains have a similar amount and the *tor1Δ* strain has a lower ~~less~~ amount. The *tor2*(K12) strain, which has mainly Tor1-TORC1, has a different phenotype from the wildtype, which has both of Tor1-TORC1 and Tor2-TORC1, e.g. phenotypes for TORC1 inhibitor and at different pH condition. Therefore, in any case, we concluded that the function of Tor1-TORC1 is different from that of Tor2-TORC1.

2) The authors wrote that “Tor2(K12) mutant largely loses its ability to form a Tor2-TORC1complex with Kog1” and that “the K12 mutant maintained sufficient Tor2- TORC2 forming ability to function as TORC2” based on the data of Figure 4A, B. However, the data show that K12 appears to have retained a bit of affinity with Kog1 and also lost a bit of affinity with Avo3. Repeat the experiments and quantify the data and discuss the impact of slightly remaining interactions and slightly decreasing interactions on the phenotypes.

As the reviewer pointed out, Tor2(K12) has a weak binding ability with Kog1 (Figure 4A, lanes 4 and 5), and the interaction of Tor2(K12) with Avo3 is weaker than that of the wild-type Tor2 (Figure 4B, lanes 2 and 3). We repeated the experiments to confirm the reproducibility of these results. We have replaced Figure 4A and 4B to improve our manuscript in this revision. We have also quantified the HA-Tor2 bands and showed that the interaction efficiencies of Tor2(K12) with Kog1 and Avo3 are 34 % ± 10 (n = 4) and 70 % ± 20 (n = 3), respectively, compared to those of the wild-type Tor2. However, the cell-based assay clearly showed that the remaining Tor2(K12)-TORC1 complex cannot function as TORC1 and that Tor2(K12)-TORC2 fulfills the function of TORC2 (Figure 3C). The in vivo kinase assay of Tor2(K12)-TORC2 (Figure 4F) and F-actin staining (Figure 4G) also support this conclusion. Furthermore, the difference in sensitivity to rapamycin, which only inhibits TORC1, in the Tor2(K12) mutant (Figure 5A) also strongly suggests that Tor2(K12) specifically affects the function of TORC1.

3) Provide molecular weight marker information for the blotting data of Figure 4.

We have described the information of the molecular weight markers information at Fig. 4A and B.

© 2024. Published by The Company of Biologists under the terms of the Creative Commons Attribution License (https://creativecommons.org/licenses/by/4.0/).

4) At line 75, "the function of Tor2-TORC1 function", delete the second "function".

Thank you, we have corrected it.

Reviewer 2 Advance Summary and Potential Significance to Field:

This manuscript entitled "Structure-based engineering of Tor complexes uncovers different roles of two types of yeast TORC1s" by Kamada et al. uncovered difference of Tor1 and Tor2 in TORC1 complex. Based on computer modelling, they introduce the mutations in Tor2 that prevent the assemble with TORC1 bun not with TORC2. By using these mutants, they showed that the cell with TORC1 either only containing TOR1 or TOR2 showed different phenotypes in rapamycin sensitivity and replicative and chronological lifespans. These suggest TOR1 and TOR2 provide different contribution in TORC1 function.

This is interesting study based on cutting edge protein engineering regarding multiprotein complex. In spite of crucial role of TORC1 in yeast, the difference between Tor1 and Tor2 had been overlooked. This study succeeded in dissection of the difference using protein engineering. Basically, the analysis was clearly conveyed, but some critical controls are missing.

We sincerely appreciate your comments. We have conducted various experiments to improve our manuscript according to your suggestions. Line-by-line responses are described below.

Reviewer 2 Comments for the Author:

1. Fig 4A. Which band is Flag-Kog1 especially in Total lysate? Negative control without expression of Flag-Kog1 is needed. It is not appropriate to cut the blot nearly at the bottom band in IP. Is the bottom band Kog1?

We appreciate the reviewer's comment. In Figure 4A of the original manuscript, it is difficult to detect Flag-Kog1 in total lysates. Therefore, we have repeated the experiment using a more concentrated lysate sample (Figure 4A in the revised manuscript). We have also added the negative control with untagged Kog1 (Figure 4A, lane 13). However, the repeated experiments still do not provide the clear band of Flag-Kog1 in total lysates. Unfortunately, we found that non-specific bands overlapped with Flag-Kog1. Moreover, Flag-Kog1 would not be stable in the lysate buffer and the concentration in lysates is probably much lower than in the immunoprecipitated samples. When immunoprecipitated, Flag-Kog1 is correctly detected as a band, while this band disappears in the negative control without the Flag tag (Figure 4A, lane 7), confirming that the bands correspond to Flag-Kog1. The lower band in the immunoprecipitated samples would be a cleavage product of Flag-Kog1 during purification.

2. Fig4B. Likewise, which is the Avo3-Flag band in Total lysate? Negative control is needed.

As the immunoprecipitation experiment of TORC1 in Figure 4A, we performed the experiment of TORC2 with higher concentrated lysate samples, together with the untagged Avo3 strain as a negative control. In Figure 4B of the revised manuscript, we were able to detect the Avo3-Flag in total lysates.

3. Fig 4D. There seem to exist subtle difference in Atg13 and Sch9 phosphorylation in tor1D cells. It is possible as Tor1 is a major component in TORC1 than Tor2. If it is true, it will affect the interpretation of in vivo data. More detailed evaluation is required in this figure. For example, how about the activation by re-addition of nutrient after starvation?

In this revision, Fig 4D was renamed to Fig. 4E. Following the reviewer's suggestion, we re-evaluated Atg13 phosphorylation in more detail by re-addition of nutrient after starvation (Figure S2A). While Atg13 phosphorylation was recovered rapidly in the wild-type and *tor2*(K12) strains, the recovery of *tor1Δ* strain was much slower. This could indeed be due to the loss of Tor1-TORC1. However, as mentioned in our response to the reviewer1, points 1 and 2, our observations suggest that Tor2-TORC1 has distinct function(s) from Tor1-TORC1. We would like to further elucidate the difference between them in more detail in the future work by performing relevant studies. We have revised our manuscript (p.9, lines 180 and 181) and added Figure S2A in the Supplementary

© 2024. Published by The Company of Biologists under the terms of the Creative Commons Attribution License (https://creativecommons.org/licenses/by/4.0/).

Information.

4. Fig 4E Negative control in which TORC2 activity is suppressed is required to evaluate the degree of phosphorylation.

In this revision, Fig 4E was renamed to Fig. 4F. It is difficult to prepare the negative control that completely suppresses TORC2 activity because TORC2 is only tor2-TORC2 and *TOR2* is essential gene.

5. Fig 4F. Again, negative control in which TORC2 activity is suppressed and actin organization is perturbed is required. Otherwise, readers cannot evaluate these images.

In this revision, Fig 4F was renamed to Fig. 4G. It is difficult to prepare the negative control by the same reason for the previous comment.

6. Fig 5A. The result that K12 is more sensitive to rapamycin than tor1D is interesting and important in this study. This data should be presented in more comprehensive manner using different dilution series of rapamycin to clearly show the difference. Furthermore, more detailed is required regarding discussion rapamycin mediated inhibition and the structure modified in this study.

We observed this phenotype using different rapamycin dilution series. Our result was reproduced as shown in Fig. S2C; the *tor2*(K12) strain is more sensitive to rapamycin than the *tor1Δ* strain. This result indicates that Tor1-TORC1 has a distinct function from Tor2-TORC1. There are several possible reasons for this phenotype. For instance, Tor1-TORC1 may have a higher affinity for rapamycin. Another possibility is that the role(s) of Tor2-TORC1 in the TORC1-mediated signaling pathway may not be fully equivalent to that of Tor1-TORC1. To clarify the mechanisms underlying the difference in rapamycin sensitivity, further studies such as solving the higher resolution structures of the *S. cerevisiae* TOR complexes and elucidating the TORC1 signaling network, which are beyond the scope of this study. We have added several sentences in the revised manuscript during the discussion session (p. 12, lines 249-252).

7. 'Different roles' in title is over interpreted. Some tone down is required.

As you mentioned, that would be over interpreted, because we have a lot more challenges to uncover the difference in roles. We changed our title to "Structure-based engineering of Tor complexes uncovers that two types of yeast TORC1 produce distinct phenotypes".

<u>Second decision letter</u>

MS ID#: JOCES/2023/261625

MS TITLE: Structure-based engineering of Tor complexes uncovers that two types of yeast TORC1 produce distinct phenotypes

AUTHORS: Yoshiaki Kamada, Chiharu Umeda, Yukio Mukai, Hokuto Ohtsuka, Yoko Otsubo, Akira Yamashita, and Takahiro Kosugi

ARTICLE TYPE: Research Article

I am happy to tell you that your manuscript has been accepted for publication in Journal of Cell Science, pending standard ethics checks.

<u>Reviewer 1</u>

Advance summary and potential significance to field

© 2024. Published by The Company of Biologists under the terms of the Creative Commons Attribution License (https://creativecommons.org/licenses/by/4.0/).

The fact that the authors succeeded in obtaining strains containing only each of the similar complexes (Tor1-TORC1 and Tor2-TORC1) in a very well thought-out manner and revealing the differences in function for the first time is highly commendable, especially with regard to the comparison of the functions of similar complexes that are usually abandoned in analyses. The fact that the same method can be used to solve similar problems in other systems is also very useful. It is also commendable that the strategies made in this paper can be applied to other systems with similar problems. Therefore, this manuscript will be of interest to a wide range of researchers in cell biology.

Comments for the author

The authors have addressed all of my concerns.

Reviewer 2

Advance summary and potential significance to field

The authors responded appropriately to my concerns.

Comments for the author

Now the paper is suitable for publication.

© 2024. Published by The Company of Biologists under the terms of the Creative Commons Attribution License (https://creativecommons.org/licenses/by/4.0/).
